# Case report: Fetomaternal hemorrhage—a lethal case and clinical insights for perinatal practice

**DOI:** 10.3389/fped.2026.1804851

**Published:** 2026-05-20

**Authors:** Tong Liu, Shaoqing Guo

**Affiliations:** 1 Department of Pediatrics, The First Affiliated Hospital of Xiamen University, School of Medicine, Xiamen University, Xiamen, Fujian, China; 2 Pediatric Key Laboratory of Xiamen, Xiamen, Fujian, China

**Keywords:** fetomaternal hemorrhage, neonatal resuscitation, neurological outcome, reduced fetal movements, severe anemia

## Abstract

**Background:**

Fetomaternal hemorrhage (FMH) is a pathological process that can cause severe perinatal complications. Its clinical manifestations are often insidious, which makes it prone to missed diagnosis. It can occur in low-risk pregnancies without identifiable risk factors, posing significant challenges to obstetric and neonatal clinicians. This case report aims to highlight the prenatal warning signs, diagnostic workflow, and management pitfalls of acute massive FMH, providing actionable insights for clinical practice.

**Case Presentation:**

A neonate born at 34^+1^ weeks exhibited severe anemia, metabolic acidosis, and shock. Despite immediate resuscitation efforts and comprehensive multi-organ support, the infant deteriorated into fatal multi-organ failure within 25 h. A maternal Kleihauer–Betke test confirmed the diagnosis, estimating a fetal blood loss of 175 mL (approximately 85.8 mL/kg). Placental pathology revealed only focal villous edema.

**Conclusion:**

FMH is an insidious perinatal emergency that demands heightened vigilance even in low-risk pregnancies. Reduced fetal movements, when combined with abnormal middle cerebral artery peak systolic velocity and cardiotocography findings, should prompt immediate obstetric evaluation. Timely multidisciplinary intervention (obstetric emergency delivery and neonatal resuscitation) and adherence to standardized diagnostic-therapeutic pathways are essential to mitigate adverse outcomes. This case provides valuable clinical insights for the early identification and management of massive FMH, particularly in pregnancies without preexisting risk factors.

## 1 Introduction

Fetomaternal hemorrhage (FMH) refers to the pathological passage of fetal blood into the maternal circulation before or during delivery (1). Its incidence is approximately 3 per 1,000 pregnancies (2). FMH has an insidious clinical presentation and requires multidisciplinary collaboration, often leading to delayed diagnosis and fragmented management. Up to 40% of severe FMH cases may be missed (3), resulting in adverse perinatal outcomes, including perinatal asphyxia, neonatal death, severe brain injury, and long-term neurological impairment (4).

We present a case of neonatal death due to acute massive FMH. We conducted a thorough literature review and systematically explored the risk factors, diagnostic approaches, treatment strategies, and prognostic assessments of FMH. This report provides actionable insights for perinatal care teams to optimize FMH management.

## 2 Case presentation

### 2.1 Maternal history

A 28-year-old primigravida (G1P0) with blood type A (Rh-positive) underwent regular prenatal checkups without abnormalities detected (e.g., gestational hypertension, gestational diabetes, or fetal growth restriction). She had no history of blood transfusions, miscarriages, or prior pregnancies. Blood tests showed no maternal anemia [hemoglobin (Hb) 12.1 g/dL, platelet count 213 × 10^9^/L, white blood cells 11.03 × 10^9^/L]. She reported reduced fetal movements for 48 h (2 days). Ultrasound and cardiotocography were performed within 30 min upon admission. Ultrasound examination revealed a fetal middle cerebral artery peak systolic velocity of 117 cm/s (2.34 MoM), markedly reduced diastolic flow, and a biophysical profile score of 3, whereas cardiotocography demonstrated a flat fetal heart rate tracing at baseline. The emergency cesarean section was completed within 45 min after confirmation of abnormal fetal status. The amniotic fluid was clear at delivery. No macroscopic abnormalities (e.g., abruption, infarction, or chorioangioma) were noted at delivery.

### 2.2 Neonatal condition

**Figure 1:**
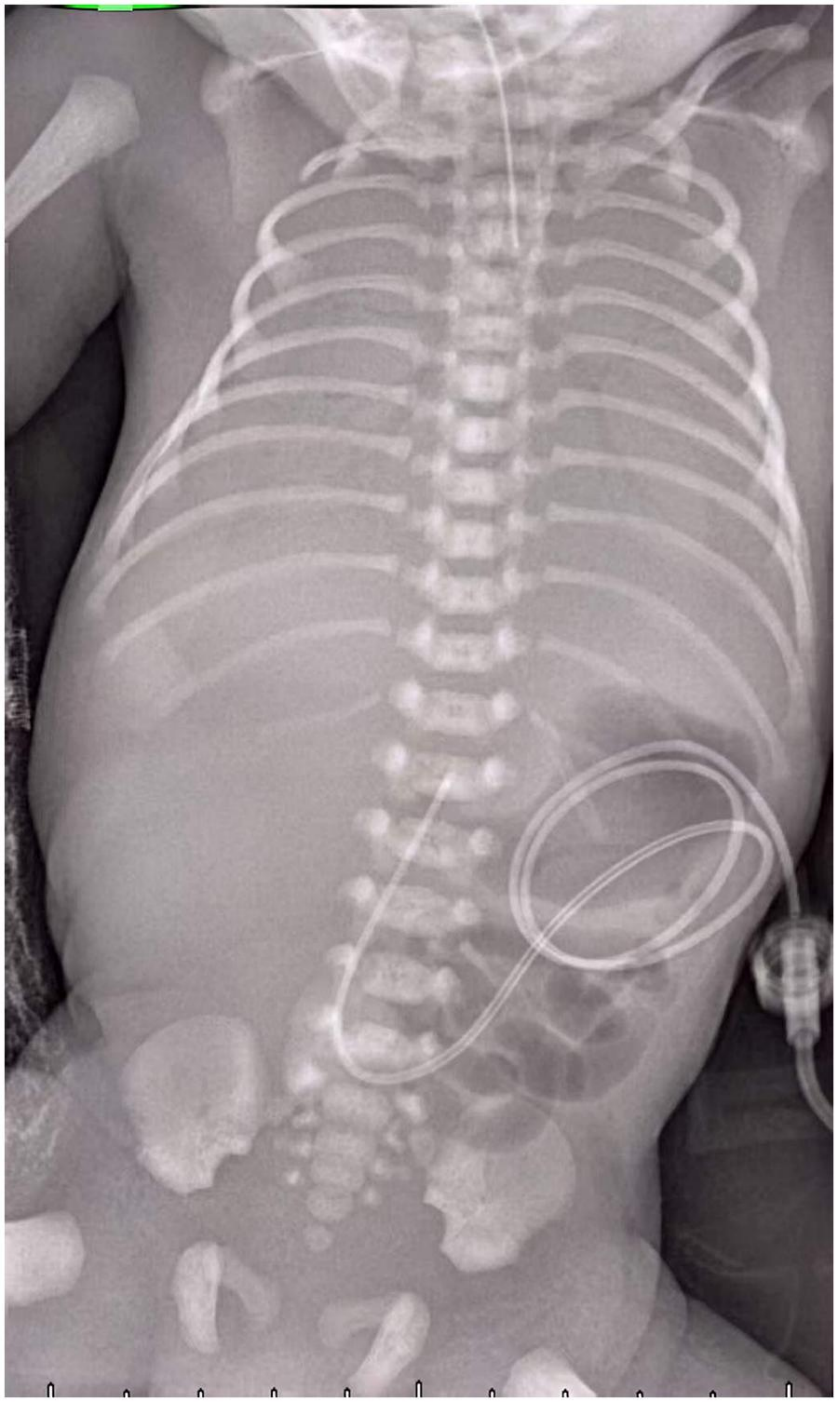
Anteroposterior chest-abdominal radiograph of the neonate showing an umbilical venous catheter and diffuse bilateral pulmonary opacification (“white lung”), consistent with severe neonatal respiratory distress syndrome.

A male infant (G1P1) was delivered by emergency cesarean section at 34^+1^ weeks due to fetal heart rate decelerations. Birth weight was 2.04 kg, length was 45 cm, and head circumference was 31 cm. Apgar scores were 6 at 1 min (deductions for respiration, muscle tone, and color), 8 at 5 min (color deduction), and 8 at 10 min (color deduction). The infant's blood group was A, Rh (D) positive, and the red blood cell (RBC) antibody screening result was negative. Respiratory distress occurred immediately after birth and necessitated endotracheal intubation and T-piece resuscitation. Immediate blood gas analysis revealed metabolic acidosis and extreme anemia (Table 1). An umbilical venous catheter was placed (Figure 1), and urgent blood product support was requested from the blood bank.

**Table 1:** Arterial blood gas (ABG) and complete blood picture (CBP) at birth.

ABG		CBP	
pH	7.282	White blood cells (×10^9^/L)	10.84
Base excess (mmol/L)	−12	Nucleated RBC (×10^9^/L)	35.08
Lactate (mmol/L)	12.8	Nucleated RBC (%)	323.6
PO_2_ (mmHg)	48	Platelet (×10^9^/L)	58
PCO_2_ (mmHg)	30.1	Hb (g/dL)	4.5
HCO_3_ (mmol/L)	14.2	HCT (%)	12.1

Investigations done at admission (ABG and CBP).

PCO_2_, partial pressure of carbon dioxide; PO_2_, partial pressure of oxygen; HCO_3_, bicarbonate.

### 2.3 Clinical course

The infant received packed RBCs (PRBC) (15 mL/kg) at 54 min of life and sodium bicarbonate (6 mL) for acidosis correction. Exogenous pulmonary surfactant (200 mg/kg) was also instilled via the endotracheal tube. Abdominal/head ultrasound and chest radiography were performed urgently, revealing no evidence of internal hemorrhage.

At 2 h of life, Hb and hematocrit increased to 7.1 g/dL and 21%, respectively. However, respiratory failure and circulatory instability persisted. Chest radiography revealed a “white lung” appearance (Figure 1) consistent with neonatal respiratory distress syndrome. Bedside echocardiography demonstrated global cardiomegaly, with preserved left ventricular systolic function (ejection fraction 57%) and normal diastolic function. A patent ductus arteriosus (3.4 mm) with continuous bidirectional shunting and a patent foramen ovale (4.5 mm) with bidirectional flow were identified, along with mild mitral and tricuspid regurgitation. No definitive evidence of pulmonary hypertension was found; the cardiomegaly was secondary to the hemodynamic stress of massive FMH and severe hypovolemic shock. Treatment was adjusted to include inhaled nitric oxide (20 ppm) and milrinone to reduce pulmonary vascular resistance, plus dopamine and dobutamine for cardiac support. Stress-dose hydrocortisone (2 mg/kg every 6 h) was administered to maintain blood pressure.

A second PRBC transfusion (15 mL/kg) was given at 5 h of life, raising the Hb to 10.8 g/dL.

High-frequency oscillatory ventilation was initiated at 12 h of life owing to severe hypoxemia and respiratory distress, with the following settings: fraction of inspired oxygen 100%, mean airway pressure 12 cmH_2_O, frequency 10 Hz, and amplitude 30 cmH_2_O. The oxygenation index was calculated as 25, consistent with severe respiratory failure.

By 24 h of life, the neonate progressed from oliguria to anuria with rapidly worsening renal function (creatinine 156 μmol/L), prompting peritoneal dialysis. Seizures were confirmed by amplitude-integrated electroencephalography showing a discontinuous background consistent with hypoxic-ischemic encephalopathy secondary to perinatal asphyxia. Seizures were controlled with intravenous phenobarbital and midazolam; levetiracetam was not used. Despite the continuous infusion of multiple vasoactive agents (dopamine, dobutamine, epinephrine, and norepinephrine), circulatory function progressively deteriorated. Heart rate and oxygen saturation steadily declined. Despite 30 min of chest compressions, repeated epinephrine boluses, and continuous vasoactive infusions, heart rate and blood pressure failed to recover. The neonate was declared dead at approximately 25 h of life. The family declined a postmortem examination.

### 2.4 Laboratory and ancillary investigation

The maternal postpartum Kleihauer–Betke test (performed within 6 h of delivery) revealed a fetal RBC proportion of 3.5%, corresponding to an estimated fetal blood loss of approximately 175 mL. No red cell fragmentation or spherocytes were observed on the peripheral blood film that was available for review. All screening test results for congenital infections (including toxoplasmosis, rubella, cytomegalovirus, herpes simplex, and parvovirus B19) were negative, as was the result of the direct Coombs test. Placental pathology revealed focal villous edema without any other structural abnormalities.

## 3 Discussion

FMH is closely associated with placental barrier disruption. Known etiologies and risk factors of FMH can be categorized into placental abnormalities (e.g., chorioangioma, placental abruption, and placenta previa), umbilical cord anomalies (e.g., single umbilical artery and true knot), maternal factors (e.g., abdominal trauma, invasive procedures such as amniocentesis or external cephalic version, and excessive uterine contractions), and fetal conditions (e.g., fetal growth restriction) (5, 6). No clear risk factors were identified in our case, indicating that FMH can occur in low-risk pregnancies. Notably, the absence of identifiable risk factors does not exclude the possibility of massive FMH.

FMH diagnosis depends on the clinician's awareness (7) and requires the integration of clinical assessment with multimodal testing. Key diagnostic methods and considerations are listed in Table 2 (6, 8–10). The outcome in the present case was multifactorial. One notable factor that substantially contributed to the severe clinical course and fatal outcome was the delayed presentation following reduced fetal movements. Moreover, acute and massive fetal blood loss (approximately 85.8 mL/kg) was observed, which is independently associated with high mortality even with timely intervention.

**Table 2:** Multimodal diagnostic methods for FMH.

Method	Core purpose/basis	Key indicator/note	Key considerations/limitations
KB Test (6)	Detect fetal RBC in maternal blood via acid elution	Standard confirmatory test for estimating fetal blood loss	Semi-quantitative; results require clinical correlation
Flow cytometry (8)	Quantify fetal Hb-positive fetal RBC	Higher accuracy, especially for minimal FMH	More equipment-dependent; not universally available
Doppler ultrasound assessment (MCA-PSV) (8)	Non-invasive assessment of fetal anemia	≥1.5 MoM suggests moderate to severe anemia	Has inherent lag time; operator and gestational age dependent
Maternal serum alpha-fetoprotein (8)	Supportive biomarker	Elevated levels may indicate massive FMH	Non-specific
Umbilical artery blood gas analysis (9)	Rapid intrapartum assessment of acidosis and anemia	Guides immediate neonatal resuscitation	Only available at delivery; indicates consequence, not cause
Reticulocyte count (10)	Differentiate acute vs. chronic FMH	≥7% suggests chronic FMH; normal count suggests acute FMH	Requires interpretation in clinical context

KB, Kleihauer–Betke; MCA-PSV, middle cerebral artery peak systolic velocity.

The clinical manifestations of FMH depend on the volume and rate of hemorrhage (11). Acute FMH often causes acute fetal decompensation, severe perinatal hypoxia, or intrauterine death, presenting at delivery with pallor disproportionate to the degree of asphyxia, bradycardia, poor perfusion, and shock that is refractory to routine resuscitation. In cases of chronic FMH with slow, gradual blood loss, the fetus is able to activate compensatory mechanisms, including enhanced erythropoiesis, which may eventually lead to the development of fetal hydrops (12).

The clinical course of this case was catastrophic—profound shock, severe anemia (Hb 4.5 g/dL, Hct 12%), and multi-organ failure within 25 h. Considering this, the cord blood gas profile showed a notable discrepancy: the umbilical cord arterial pH was only 7.282, with a base excess of −12 mmol/L, suggesting only moderate metabolic acidosis. This can be explained by the rapid onset of hemorrhage. In acute massive FMH, profound hypovolemia, tissue hypoperfusion, and circulatory collapse develop so abruptly that there is insufficient time for severe acidosis to fully manifest and be reflected in cord blood gas measurements at the time of delivery (9). Therefore, a relatively mild acid-base profile does not rule out catastrophic FMH; rather, the degree of anemia, clinical signs of shock, and the Kleihauer–Betke test (6) are more reliable indicators of severity.

Reduced fetal movements and sinusoidal heart rate patterns require obstetric vigilance (13). Although reduced fetal movement has limited specificity and requires correlation with cardiotocographic changes, it is the most common and earliest warning sign of FMH. Conversely, although characteristic of FMH, sinusoidal patterns are uncommon.

Massive FMH should be considered in all cases of unexplained fetal death, severe fetal distress, non-immune hydrops fetalis, or nonhemolytic neonatal anemia. After diagnosis, placental histopathological examination is recommended along with serial human chorionic gonadotropin monitoring to exclude choriocarcinoma (14).

The management of massive FMH primarily depends on the gestational age at diagnosis, fetal condition, and maternal status (15). For fetuses <32 weeks, intrauterine transfusion is the preferred treatment to allow time for further maturation. Between 32 and 34 weeks of gestation, individualized management is necessary; fetal lung maturity must be achieved prior to delivery, with a careful decision between intrauterine transfusion and delivery. For gestational age ≥34 weeks with signs of fetal compromise, timely delivery is recommended.

The cornerstone of neonatal management is the rapid restoration of effective circulating volume and tissue oxygenation. Once massive FMH is suspected, immediate multidisciplinary intervention is essential to ensure the readiness of the neonatal team and blood product availability. Correction of hypovolemia is critical for successful resuscitation (16).

**Acute FMH with hypovolemic shock:** Immediate volume resuscitation (11) is indicated. O-negative, Rh-negative PRBC (even uncrossmatched) is preferred and administered at 10–15 mL/kg. If whole blood is unavailable, normal saline, fresh frozen plasma, or 5% albumin may be administered. In preterm infants, the infusion rate must be controlled to prevent blood pressure spikes and the risk of intracranial hemorrhage or pulmonary edema.

**Chronic FMH with severe anemia and normovolemia or hypervolemia (often with edema)** (3): Partial exchange transfusion (PET) with high-hematocrit (>60%) PRBC is recommended to rapidly improve oxygen-carrying capacity without increasing the cardiac load.

The infant presented with acute hypovolemic shock secondary to massive FMH, for which rapid volume replacement was the immediate priority. We administered two standard PRBC transfusions (a total of 30 mL/kg) at 54 min and 5 h after birth. PET was considered but not undertaken, as this procedure is time-consuming and requires careful volume calculation and controlled phlebotomy. Given the rapid, relentless clinical deterioration, and considering the infant's death at 25 h of life, the therapeutic window was extremely narrow and did not allow sufficient time to perform PET safely. Nonetheless, in similar cases where initial resuscitation leads to relative hemodynamic stability, PET may offer distinct benefits by rapidly increasing Hb while avoiding excessive volume overload (17).

To further illustrate the management strategies and outcomes in massive FMH, we summarized similar published cases in Table 3 (13, 15–21), comparing patients treated with PRBC transfusion alone vs. those treated with PET. Notably, Menéndez et al. (18) reported a neonate with chronic anemia due to FMH who developed volume overload and clinical deterioration after initial PRBC transfusion. Following PET, the infant's condition improved significantly with no residual sequelae.

**Table 3:** Similar cases with massive FMH: treatment strategies and outcomes.

Author, Year (reference)	Category	Gestational Age at Birth (Weeks)	Birth weight (g)	KB test (%)	Estimated Blood Loss (mL)	Hb at birth (g/dL)	Hematocrit at birth (%)	PRBC transfusion (mL)	PET (mL)	Hb at Post-Treatment (g/dL)	Hematocrit Post-Treatment (%)	Outcome
Naulaer et al. (20)	Chronic FMH	39	2,450	4.5	225	3.7	11.3	180 mL transfused followed by PET	390 mL O Rh-negative Whole blood	14	42	Normal at 1 year
Naulaer t al. (20)	Acute FMH	34	2,630	positive	100	5.6	18	50 mL transfused	Not done	12.6	40	Normal at 1 year
Kuin et al. (16)	Acute FMH	37	3,200	5.7	285	3.1		15 mL/kg transfused	Not done	Not report	Not report	Normal
Menéndez et al. (18)	Chronic FMH	34 + 3	2,320	3.08	152	3.4	12.4	20 mL/kg transfused prior to PET	Done with whole blood	13.5	38.8	Normal at 2 years
Miyahara et al. (19)	Chronic FMH	27	998	2.4	120	1.2	4.5	16 mL prior to PET	180 mL Whole blood	8.9	27	Normal at 18 months
Gică et al. (21)	Acute FMH	37	3,050	positive	490	3.6	12.8	90 mL transfused	Not done	Not report	32	Normal at 6 months
Laxman et al. (17)	Acute FMH	35	2,100	2.7	135	5.6	15.2	Not done	75 mL O Rh-negative PRBC	13.9	42	Normal at 6 months
Zheng et al. (13)	Acute FMH	37 + 5	3,020	positive	Not report	2.6	9	150 mL transfused	Not done	Not report	Not report	Normal
Zheng et al. (13)	Acute FMH	37	2,380	positive	Not report	5.2	18	70 mL transfused	Not done	Not report	Not report	Normal
Derme et al. (15)	Acute FMH	38 + 4	2,720	positive	114	4.3	15.3	44 mL transfused	Not done	11.8	39	Normal at 3 months
Present Case	Acute FMH	34 + 1	2,040	3.5	175	4.5	12	30 mL/kg transfused	Not done	10.8	32	Death at 25 h of life

KB, Kleihauer–Betke.

This experience highlights two key principles in the management of massive FMH (12). First, the timing and chronicity of fetal blood loss are critical determinants of clinical presentation; acute massive FMH manifests with hypovolemic shock that demands urgent volume replacement, whereas chronic FMH typically results in normovolemia or even hypervolemia (e.g., fetal hydrops). Second, in chronic FMH, following initial transfusion, close monitoring for volume overload (including respiratory distress, hepatomegaly, and worsening edema) is essential. If such signs develop, exchange transfusion or PET using high-hematocrit PRBC should be initiated promptly to optimize oxygen-carrying capacity without exacerbating cardiac preload. Therefore, a single transfusion strategy is not suitable for all FMH cases; rather, management must be individualized based on the estimated volume of fetal blood loss, the acuity of hemorrhage, and the neonate's hemodynamic and volume status.

Multiorgan supportive care includes respiratory support (e.g., mechanical ventilation and surfactants), circulatory support (vasoactive agents), renal replacement therapy (peritoneal dialysis and continuous renal replacement therapy), and neuroprotective measures tailored to the infant's clinical condition.

The outcomes of FMH correlate strongly with the severity of blood loss, timeliness of the intervention, and management of complications (1). More severe anemia is associated with a worse prognosis, and perinatal mortality increases significantly when blood loss exceeds 40 mL/kg (22). Key risk factors associated with mortality in acute massive FMH include delayed prenatal recognition, acute massive fetal hemorrhage, severe hypovolemic shock, refractory multi-organ dysfunction, and an extremely narrow therapeutic window (12). In the present case, a combination of these factors resulted in the fatal outcome despite maximal resuscitation efforts.

Studies have reported death or long-term neurological sequelae in 38%–50% of severe FMH cases (4). However, the incidence of FMH recurrence remains low. A 10-year cohort study of 143 patients with FMH found only one instance of FMH recurrence in subsequent pregnancies (23). All survivors of FMH should be enrolled in systematic long-term follow-up programs, including neurodevelopmental assessment, hearing and vision screening, and cognitive-motor function monitoring.

## 4 Conclusion

FMH is an insidious yet life-threatening perinatal emergency. This case highlights that clinicians must maintain high vigilance even in pregnancies without identifiable risk factors, with particular emphasis on reduced fetal movements as an early warning sign. This case report increases clinical awareness of FMH and provides actionable insights for early identification and management, ultimately improving perinatal outcomes.

However, this single case report has limited generalizability. Its retrospective nature and lack of autopsy constrain pathological insight. Multicenter studies are required to explore more sensitive diagnostic markers and effective interventions.

## Data Availability

The original contributions presented in the study are included in the article/Supplementary Material, further inquiries can be directed to the corresponding author.
